# Sleepiness, Neuropsychological Skills, and Scholastic Learning in Children

**DOI:** 10.3390/brainsci10080529

**Published:** 2020-08-07

**Authors:** Luigi Macchitella, Chiara Valeria Marinelli, Fulvio Signore, Enrico Ciavolino, Paola Angelelli

**Affiliations:** Department of History, Society and Human Studies, Lab of Applied Psychology and Intervention, University of Salento, 73100 Lecce, Italy; luigi.macchitella@unisalento.it (L.M.); fulvio.signore@unisalento.it (F.S.); enrico.ciavolino@unisalento.it (E.C.); paola.angelelli@unisalento.it (P.A.)

**Keywords:** sleepiness, cognition, learning, reading, writing, comprehension, mathematical skills

## Abstract

Excessive daytime sleepiness is a frequent condition among children and adolescents that may lead to several and significant daytime consequences, including impaired neurocognitive skills and scholastic performance. Here, we evaluated in one hundred and ninety-one unselected primary school children, the relationship between sleepiness and a wide range of cognitive and academic skills through a standardized neuropsychological test battery. In order to assess the statistical relationship, we performed a partial least squares path modelling, a non-parametrical approach which combined a model of paths between latent variables and the coefficients between indicators and dimensions. Results were validated through the bootstrap approach and suggest that sleepiness is not associated with all cognitive and scholastic abilities, but only with those relying on verbal abilities and complex cognitive functions (i.e., reading comprehension, oral/syntactic comprehension, spelling, and mathematic skills). Our data suggest the idea that sleepiness in children is associated mostly with “higher” (mainly verbal) cognitive function(s), while the visuospatial domain was not affected.

## 1. Introduction

Several lines of research point out that sleep may influence (both positively and negatively) neurocognitive development as well as cognitive skills and scholastic achievement in children and adolescents [1,2,3,4,5,6,7,8]. Sleepiness is the difficulty in maintaining a desired level of wakefulness or arousal [2,5], while excessive daytime sleepiness (EDS) is defined as an increased tendency to fall asleep in a setting where an individual would be expected to stay awake and alert [9]. “*Excessive daytime sleepiness represents a common but often under-recognized phenomenon in children*” [10]. Indeed, many studies reported a high rate of EDS among children and adolescents, ranging from 10% to 47% [11]. At the same time, however, EDS is underreported by parents and underdiagnosed by physicians, possibly due to lack of recognition [11]. EDS may increase risky behaviours (such as motor vehicle accidents in adolescents) and it may be associated to impaired cognitive and scholastic performance [12,13]. There are numerous and complex biopsychosocial conditions that may cause (independently, jointly or through their interactions) excessive daytime sleepiness [9,10,11,12,14]. They may be conceptualized under the following broad categories: Insufficient sleep duration (due, for instance, to poor sleep hygiene as well as biological factors; see also below), fragmented/disturbed sleep (caused by, for example, medical problems such as gastroesophageal reflux as well as sleep disorders such as sleep-disordered breathing (SDB)), circadian misalignment (e.g., delay sleep-wake phase syndrome), primary disorders that increase sleep needs (e.g., depression, narcolepsy, hypothalamic lesions) [9,11] (Morse and Kothare, 2019; Owens et al., 2020), and intrinsic development related to changes in biological systems (i.e., homeostasis and circadian systems) that regulate timing of sleep and awakening [15,16,17]. Finally, there are several “environmental and lifestyle factors” that may induce EDS, such as, for example, school starting times and staying awake until late to finish homework (that may induce a reduction of sleep duration) [18,19], excessive consumption of food and drink containing caffeine [20,21,22], pre-sleep activities (e.g., the use of television, tablet, and video games may induce both delay in bedtime and shortens sleeping hours as well as impaired sleep latency and sleep architecture) [22,23,24,25,26,27] as well as more trivial but still important factors (environmental stimuli such as noise, light, temperature during sleep time) (Morse and Kothare, 2019). Notably, sleepiness associated with insufficient sleep duration may arise from complex interactions between environmental factors (e.g., school starting time) [9] and intrinsic development related to changes in biological systems that regulate sleep time [14,17,28]

Considering that EDS is a frequent condition among children and adolescents that may have several and important daytime consequences, EDS has become an important international health and societal concern. The scientific and media attention on this problem has already influenced some public policy, school, and clinical practice. For example, pediatric health care professionals were required to inquire about sleep and the biopsychosocial causes underling EDS in their patients [11]. Moreover, considering the “environmental and lifestyle factors” that may induce EDS, Minges and Redeker stressed that “*given the public advocacy for delayed school start time, the recent policy statement from American Academy of Paediatrics to delay the start of class to 8:30 a.m. or later and some estimates that over 80 U.S. school district have already adopted later school start time*” [18]. Finally, the lockdown during COVID-19 pandemic has made EDS a very topical issue. Indeed, the lockdown has affected several habits in children and adolescents, some of which are related to sleep habits, such as an excessive use of video games, computer or mobile telephones that may increase daytime sleepiness [29,30,31,32]. For example, in Italy a 70% increase in Fortnite-gaming related internet traffic was reported [33].

What are the predictable effects of sleepiness on cognitive and scholastic performance in children and adolescents? Several reviews stressed the impact of ESD on cognition and scholastic performance in children [5,10,13]. However, in some cases, assumptions about the relationship between sleepiness and both cognition and school performances arise indirectly. Most of the studies investigated the effects of primary conditions leading to sleepiness (e.g., sleep disorders, poor sleep quality or quantity assessed, for instance, via actigraphy) on cognitive skills and scholastic achievement. Accordingly, these studies found that sleep disorders and poor sleep quality or quantity may adversely affect intelligence, executive functions, attention, visuo-motor skills, working memory, long term memory and learning, as well as the academic skills such as reading, reading comprehension, spelling, and arithmetic skills [4,34,35,36,37,38,39,40,41,42,43,44,45,46]. Furthermore, studies that experimentally manipulated sleep duration in children and adolescents, introducing some form of restrictions/deprivation, highlighted decreased cognitive performances [5,47,48,49,50,51,52] as well as increased school difficulties [5,53,54]. In sum, in many of such studies it was found that children with insufficient or impaired sleep were sleepier during the day and displayed poorer cognitive and scholastic performances. This evidence suggested a “direct” association between sleepiness and decremented cognitive and/or school skills [5,10,12,13]. Crucially, however, in many of these studies the relationship between children sleepiness and cognitive performance was taken for granted and not evaluated. For example, the study of Randazzo et al. [48] is one of the studies frequently cited in the reviews addressing the impact of sleepiness on cognition. Randazzo et al. [48] found that experimental sleep restriction induced both sleepiness and poorer cognitive performance, however the association between sleepiness and cognitive impairment was not assessed. This is an important methodological question that, in turn, suggests caution when affirming a relationship between sleepiness and cognitive/academic skills. Indeed, despite insufficient/disturbed sleep that induce both sleepiness and cognitive sequelae, it is not possible to take for granted that decremented cognitive performances are due to sleepiness. Both sleep loss and sleep disorders may affect cognition independently from sleepiness [2,39,42,51,55]. For example, Jiang et al. [51] investigated the effect of sleep restriction on both sleepiness and working memory and found that sleep loss affects both subjective sleepiness and working memory. At the same time, however, the authors failed to find a correlation between sleepiness and the working memory performance [51]. Moreover, children with SDB may exhibit impairment in executive functions, independently from their sleepiness levels [42]. In other words, their deficits may extend beyond those associated with sleepiness. Indeed, the causal mechanism of these cognitive effects remains open to debate.

Thus, it seems reasonable that, in order to support the idea that there is a relationship between sleepiness and cognition, studies assessing directly and specifically the association between sleepiness and cognitive skills are needed [44]. There are relatively few studies and data which seem to support the relationship between sleepiness and cognition and between sleepiness and scholastic achievements in different samples, i.e., non-selected population of children, healthy children, or children with sleep disorders [56,57,58,59,60,61,62,63]. However there are also some studies which failed to find any association [39,42,51]. Conflicting results regarding which of the cognitive skills is the most affected by sleepiness were also reported. Buckhalt et al [57,60,61,64] found that subjective sleepiness correlated with several cognitive functions (including intellectual ability, verbal comprehension, and working memory) and with mathematics, language, and reading achievements. Calhoun et al. [65] showed a correlation between sleepiness not only with some cognitive skills (processing speed and working memory), but also with school learning problems (e.g., failure to complete school work or the school year), as reported by parents. Notably, other data indicate that there is no association between sleepiness and working memory [51] or intelligence [65]. Similarly, inconsistent findings also regard the relationship between sleepiness and executive functions. Anderson et al. [59] found that adolescents who reported high levels of sleepiness had poor executive functioning. Moreover, Cerasuolo et al. [63] found a significant correlation between sleepiness and executive functions in children (assessed via a Go/NO GO task). Other studies, however, failed to find a correlation between sleepiness and other tests of executive function (i.e., Stroop Test and Wisconsin Card Sorting Test; Calhoun et al., 2012; Esposito et al., 2013). In summary, even if a link between sleepiness and cognitive functions was documented, data are still relatively few and results controversial. Moreover, previous studies focused mainly on some cognitive abilities (attention, executive functions, and verbal related ability such as verbal working memory and verbal comprehension), while the relationship between sleepiness and other cognitive abilities (e.g., visual and spatial abilities) was studied less thoroughly. Interestingly, taking together the sparse evidence, it seems that the association between sleepiness and verbal related tasks (e.g., phonological working memory and verbal comprehension underlying crystallized intelligence) is consistent across studies [57,65], while less evidence regarding non-verbal domains of cognition (e.g., visuo-motor integration sills) was reported.

Finally, an important note of caution in interpreting previous findings regards the procedure used for the assessment of scholastic outcomes. Almost all studies did not assess school achievements through standardized tests. For example, school learning outcomes were assessed mainly via self-, parent- or teacher- reports [56,58,61,65,66,67] or through scholastic report cards [68]. Only some studies on children suffering from sleep disorders (mainly SDB) used standardized tests and found that sleep disorders, usually associated with sleepiness, affected scholastic learning outcomes (such as spelling, reading, and arithmetic skills) [36,38,41,43]. This, in turn, suggested the hypothesis that sleepiness could affect scholastic learning. However, the primary effects of sleep disorders on scholastic outcomes deserves caution in interpreting the data, since (as stressed above) sleep disorders may adversely impact cognitive functioning independently from sleepiness (e.g., via hypoxia, sleep fragmentation, and related impact on brain restorative mechanisms [42].

The purpose of this study is to evaluate the relationship between sleepiness and several cognitive skills and scholastic learning abilities, assessed with an extensive neuropsychological battery of standardized tests and not through teachers’ and parents’ reports. We evaluated, in a large sample of unselected primary school children, the association between sleepiness and cognitive abilities, in both verbal (e.g., syntactic comprehension skill) and non-verbal (i.e., visuo-spatial ability) domains, as well as academic skills (e.g., reading decoding, reading comprehension, spelling, mathematical, and handwriting skills). Finally, we wanted to explore if sleepiness may be more related to verbal than to non-verbal domains of cognition and which subdomains may be more influenced. We used a very suited statistical technique, partial least squares path modelling (PLS-PM), the non-parametrical extension of structural equation models (SEM), which allows to suggest causal links between sleepiness and the various academic skills, each interdependently from the other. The additional benefit of this statistical modelling is the combination of dependence and prediction through regression coefficients between latent variables, and interdependence, through the different items of the tests synthetized in one or more common dimensions by performing a factor analysis.

## 2. Methods

### 2.1. Participants

One hundred and ninety-one children (91 female and 100 male), ranging in age from 7.8 to 11.2 years (average age: 9.61 ± 0.90), were recruited from three primary schools in the south of Italy. In particular, 60 3rd grade children (32 F, 28 M, average age: 8.56 ± 0.32), 57 4th grade children (29 F, 28 M, average age: 9.50 ± 0.32), and 74 5th grade children (30 F, 44 M, average age: 10.56 ± 0.31) participated in the study. All children performed within the norm at an intelligence test [69].

With the guide of an expert psychologist, all children completed the questionnaire assessing sleepiness and then were evaluated through several neuropsychological tests (see below). Participants were tested individually in the morning in a quiet room during school time. The tests were administered through a pseudo-random sequence across participants.

The parents were informed of the research activities and authorized their child’s participation by signing the appropriate informed consent. The study was conducted according to the principles of the Helsinki Declaration and was approved by the school authorities when it started. It was also approved by the Ethic committee of Psychological Research of the Department of Hystory, Society and Human Studies -University of Salento (Prot. 101206 -29th July 2020).

### 2.2. Sleepiness Measures

We evaluated sleepiness through the Pediatric Daytime Sleepiness Scales [56]. PDSS is a self-reported likert-type questionnaire that measures daytime sleepiness thought related behaviours (e.g., falling asleep or getting drowsy during class periods or homework; difficulty getting up in the morning) in scholastic populations, with possible scores ranging from 0 to 32. Higher PDSS scores indicate greater daytime sleepiness.

PDSS has a high (0.80) internal consistency (assessed through the split-half) and acceptable factor loadings [56]. Moreover, the PDSS scores correlate with total sleep time and sleep disorders [56,58,70].

### 2.3. Neuropsychological Assessment

#### 2.3.1. Reading and Comprehension Skills

The participants’ reading level was assessed through a standard reading achievement test widely used for Italian children [71]. The MT consists of a series of meaningful texts (short stories taken from children books) of increasing difficulty from Grade level 1 to 5: Syntactic and semantic complexity increases progressively; the letter size decreases (from a medium width of approximately 3.8 to 1.6 mm), and the number of words increases (from 66 to 281 words) progressing from Grade 1 to Grade 5. Children were asked to read a single text depending on their grade and school-year period. Each story was printed in black on a white cardboard. Participants were asked to read the text aloud within a 4-min time limit. Reading speed (number of syllables read/sec) and accuracy (number of errors, adjusted for the amount of text read) were considered. A second passage evaluated reading comprehension: Children read it without a time limit and responded to 10-multiple-choice questions. Children could check the text while answering so that memory load did not affect performance. Correct answers were collected.

#### 2.3.2. Spelling Skills

The participants’ spelling abilities were tested with a standard dictation test [72]. We considered two sections, both requiring the application of transcribing processes based on one-sound-to-one-letter correspondence:

Section A: Regular words with complete one-sound-to-one-letter correspondence (N = 70). Words were selected with different sources of phonetic-phonological complexity: (i) Words made up of continuant sounds only (fricative, liquid, or nasal consonants) vs. words also containing non-continuant (plosive) consonants; (ii) words made up only of consonant-vowel (CV) syllables vs. words also containing consonant clusters and doubled consonants; (iii) disyllabic vs. polysyllabic words. The presence of different sources of phonetic-phonological complexity influences both segmentation and identification of phonemic string to be converted (for instance, continuant phones are, by nature, easier to segment, and hence to identify, than non-continuant phones).

Section B (that in the test corresponds to Section D): Pseudowords—with one-sound-to-one-letter correspondence (*N* = 25). Items were controlled for different sources of phonetic-phonological complexity, as were words in Section A: (i) Continuance of sounds (non-words with continuant vs. non-continuant consonants); syllabic structure (non-words with consonant-vowel (CV) syllables vs. non-words also containing doubled consonants; and length (disyllabic vs. 3–4 syllable non-words). Similarly to Section A, phonetic/phonological variables are introduced in order to account for variables influencing acoustic-to-phonological analysis that is preliminary to an effective phonological-to-orthographic conversion procedure.

Words and pseudowords were given in separate blocks and in a single quasi-randomized order. The examiner read each item aloud in a neutral tone. The children were asked to repeat each item, before writing, by spelling it down (so that the examiner could ensure that they had perceived the item). They were permitted to write in either capital or lower case letters. No feedback was provided on the accuracy of the written response. The number of stimuli spelled correctly in each section was computed. Self-corrections were accepted.

#### 2.3.3. Mathematical Skills

The AC-MT battery [72] was used to assess the students’ mathematical skills. AC-MT is a battery composed by several subtasks that are derived from the neuropsychological model of number processing and calculation [73,74,75]. The subtests used in the present work are described below:

- Computation. This subtest assesses the child’s ability to complete written computational operations (additions, subtractions, multiplications, and divisions).
- Number ordering. This task requires understanding semantics of number and thus evaluate number sense. A series of four numbers are presented, and the child must be able to place them in the correct order (from greatest to smaller one, and vice versa).
- Number spelling. This task assesses students’ ability to lexical retrieval as well as to elaborate the syntactic structure of a number. Students listen to six numbers and then are asked to spell them.
- Arithmetical Facts subtest. This task is used to investigate if children have stored arithmetical facts and were able to retrieve automatically the results of basic and simple operations from the memory. Children are asked to recall several arithmetic facts, each one within 5 s of time.

In all subtests, correct answers were collected.

#### 2.3.4. Syntactic Comprehension Skill

Syntactic comprehension test [76] assesses oral comprehension and in particular the ability to identify the stimulus listened between syntactic distractors. Participants had to choose one picture among four alternatives that represented the sentence read by the examiner. Correct answers were collected.

#### 2.3.5. Handwriting Speed

The Concise Assessment Scale for Children’s Handwriting [77] is a standardized norm-referenced test to assess handwriting skills. Children were required to copy in their usual style a standard text on an unlined A4 sheet in a maximum 5 min (for slow writers, children had to spell at least the first five sentences) Copying speed (BHK-speed) was determined by counting the number of letters spelled in 5 min.

#### 2.3.6. Visuospatial Working Memory

Visuospatial working memory (VSWM) was assessed through the *Visual Pattern test, Active Version (VPTA)* of the Visuospatial Working Memory Test Battery [78]. This test assesses the active simultaneous component of visuo-spatial working memory [79,80]. Note that active processing requires the ability to integrate and modify previous stored visuo-spatial information. The task requires memorizing a pattern of positions in a matrix; after that the children are required to reproduce it on an empty matrix, but shifting the original pattern one line below. The total accuracy was scored.

#### 2.3.7. Sustained and Selective Attention

The bell test [81] evaluates sustained and selective attention. The test consists of four different sheets, each of which contain 35 bells among many other stimuli (animate and inanimate objects, such as houses, trees, fish, horses, etc.) of the same size and orientation in space. The task consists of finding and ticking only the bells within a time limit (2 min per sheet). We calculated the total number of bells identified in the first 30 s on each sheet of paper (selective attention score).

#### 2.3.8. Visuo-Spatial Constructive Skills

The Rey-Osterrieth complex figure test [82] is a neuropsychological test extensively used in neuropsychological research and clinical practice. Through the use of different scoring systems, the ROCF task allows evaluating several cognitive abilities, including visual memory, visuo- spatial constructional ability, as well as executive functions such as planning strategies [83,84,85]. Children were asked to copy a complex geometrical figure as accurately as possible. The score was computed according to Ferrara-Mori [86].

## 3. Data Analyses

### Partial Least Squares Path Modelling (PLS-PM)

For each test, raw scores were converted to z scores according to proper standard reference data. Preliminarily, we looked for differences in the rate of subjective sleepiness as a function of gender and school age. Non-parametrical comparisons of mean, or Welch’s Test and ANOVA (due to the different standard deviations of the groups), were performed with gender (two levels: Male vs. female) and school age (three levels: 3rd, 4th, 5th graders) as independent variables and values of sleepiness as dependent ones.

In order to test the relationship between sleepiness and the various academic skills, we performed a partial least squares path modelling [87], a multivariate variance based on the structural equation modelling technique whose application can be found in business, management, and in social sciences, as well as recently in psychology [88,89,90]. Structural equation modelling (SEM) involves the analysis of multiple variables taken into account simultaneously and represents a second-generation technique, due to the sophisticated methods used. This technique allows modelling latent variables based on behavioural research, composite constructs, and different measurement scales. This technique allows modelling latent variables based on behavioural research, composite constructs, and different measurement scales. The aim of SEM is, therefore, to investigate the relations between directly non-observable variables (i.e., latent variables), which are, in turn, measured by indicators (i.e., manifest variables), provided by scales. We used PLS-SEM, which is a primarily explorative technique, because it does not imply assumptions on distributional data, nor on sample size. Furthermore, PLS-SEM is a robust method when there are missing values (at a reasonable level) within data. The model in PLS-SEM can be defined by using constructs formed by single and multi-item measures. Finally, PLS-SEM can incorporate formative and reflective models [91] minimizing the amount of unexplained variance and is more efficient, since the convergence takes place after few iterations [92].

**Figure 1:**
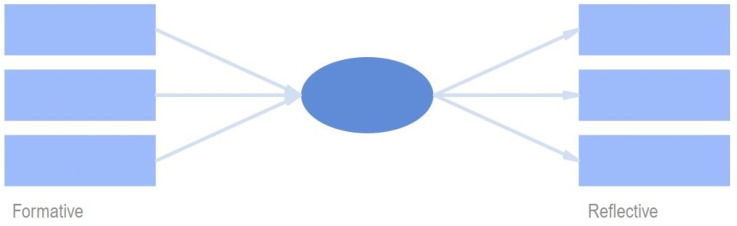
Formative or reflective blocks.

The aim of this research was to inquire the existing relations between sleepiness and other neuropsychological abilities [93], as mathematical skills, attention, spelling, oral and reading comprehension, reading, visuo-constructive skills, visuo-spatial working memory, and handwriting. In order to achieve this objective, we performed a PLS-PM model with ten latent variables measured through 18 different indicators, as reported in Table 1. We used the reflective approach to define the blocks of indicators (Figure 1).

In the reflective approach (right side of Figure 1), manifest variables are supposed to be caused by the latent variables. In the formative way (left side of Figure 1), instead, the latent construct is constituted by its indicators. The difference between the two approaches is based on causal-effect relationships between the indicators and the constructs. In a reflective block, a change in the latent variable corresponds to a change in its indicators.

The goodness of measures reliability was assessed through three different methods, Cronbach’s alpha, Dillon-Goldstein’s rho, and the eigenvalues criterion. Cronbach’s alpha and Dillon-Goldstein’s rho values must be bigger than 0.70 in order to be considered good indices of reliability. In terms of eigenvalues, the first one must be bigger than the value one while the second smaller than one. Latent variables measured by only one indicator have a subsequent value of 1.

With regards to the outer model, guidelines [87,92] affirmed that indicators must have a loading of at least 0.708 to be considered good reflective blocks of latent variables. This precondition is fundamental to explain an account of variance at least at 50%, or communality, which is the variation in an item (i.e., a test) explained by the construct (variance extracted from the item).

We determined the average variance extracted (AVE): This is an index which allows to evaluate the amount of variance of the indicators explained by the latent variables. A good AVE index is more than 0.50.

## 4. Results

The explorative analysis on differences in sleepiness due to gender distribution showed no significant effect (more specifically MEAN _FEMALE_ = 13.8 (SD = 6.55) and MEAN _MALE_ = 13.9 (DS = 5.49), ∆ = 0.0839; *p*-value = 0.933). Similarly, no significant differences emerged as a function of school age (MEAN _THIRD GRADE_ = 15.5 (SD = 6.13); MEAN _FOURTH GRADE_ = 13.6 (SD = 5.58), MEAN _FIFTH GRADE_ = 12.7 (SD = 5.94), *p*-value = 0.062). Overall, we can assume that, in our sample of primary school children, gender and age did not modulate levels of subjective sleepiness.

**Figure 2:**
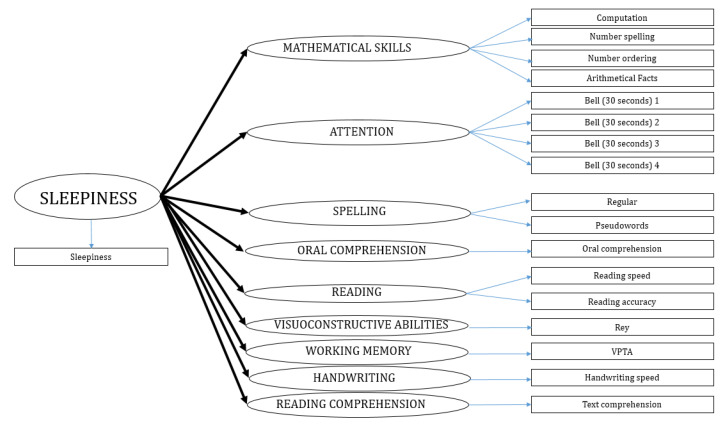
The inner and outer model.

Partial least squares path modelling (PLS-PM) was performed through the plspm package [87] by using the R Studio software (version 1.2.5033). The hypotheses were tested by performing a PLS-PM model, as shown in Figure 2.

Table 2 reported results of goodness of measures reliability. All latent variables present good Cronbach’s alpha and Dillon-Goldstein’s rho indexes. Only the latent construct *mathematical skills* had a slightly lower measure of 0.70 (0.69). Nevertheless, other indices were acceptable, therefore we considered all the non-observable constructs as appropriately reliable.

With regards to the outer model, we found only two indicators which did not reach a loading of 0.70, *computation* and *number spelling* on *mathematical skills* (respectively 0.69 and 0.68) but their measures were very near to that threshold, so we included them in our model without losing quality (Table 3).

**Figure 3:**
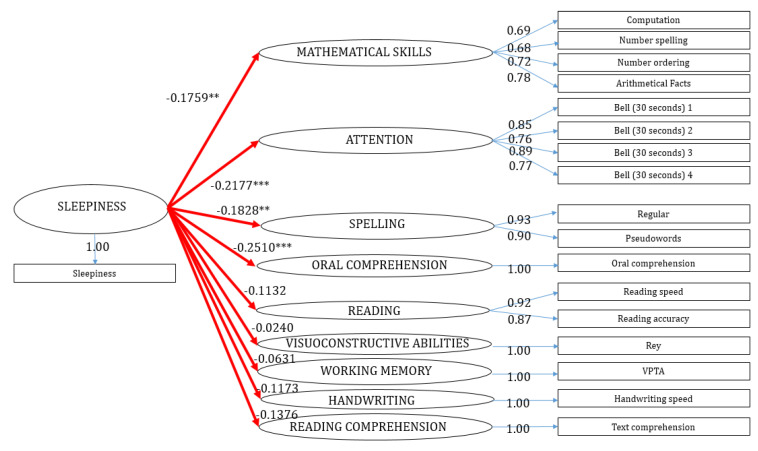
The model with path coefficients and loadings. ** *p*-value < 0.05, *** *p*-value < 0.001.

Table 4 presented cross-loadings values. These measures are important to identify indicators with the higher correlation with the latent variable, which should be the one to which they are associated. The cross-loadings approach assesses the discriminant validity. In our study, the biggest correlations of the indicators corresponded to their non-observable constructs. The final model is presented in Figure 3.

Regarding the AVE index, we found that AVE _SLEEPINESS_ = 1, AVE _MATHEMATICAL SKILLS_ = 0.515, AVE _ATTENTION_ = 0.672, AVE _SPELLING_ = 0.845, AVE _ORAL COMPREHENSION_ = 1, AVE _READING_ = 0.804, AVE _VISUOCONSTRUCTIVE SKILLS_ = 1, AVE _VISUO SPATIALWORKING MEMORY_ = 1, AVE _HANDWRITING_ = 1, and AVE _READING COMPREHENSION_ = 1. According to these AVE outcomes, each latent variable explains more than 50% of the variance, therefore the remaining error is less than the information explained.

Based on our results, sleepiness had negative coefficients with all latent variables used. In particular, the statistical relations were negative and significant (α = 0.05) in four causal effects, attention (β_2_ = −0.2177, *p*-value = 0.007), mathematical skills (β_1_ = −0.1759, *p*-value = 0.03), spelling (β_3_ = −0.1828, *p*-value = 0.024), and oral comprehension (β_4_ = −0.251, *p*-value = 0.002). In the other cases, sleepiness did not affect in a significant way reading-decoding (β_5_ = −0.113, *p*-value = 0.154), visuo-constructive skills (β_6_ = −0.024, *p*-value = 0.786), visuo-spatial working memory (β_7_ = −0.063, *p*-value = 0.439), and handwriting (β_8_ = −0.117, *p*-value = 0.149). The relation between sleepiness and reading comprehension is negative (β_9_ = −0.138) and not significant but *p*-value was very close to the threshold of 0.05 (*p*-value = 0.09), so this result must be considered as a borderline significant trend. In order to assess the validity of the model, we performed different resampling through the bootstrap method in path coefficients (*n* = 1000). Results were confirmed in terms of polarity and significance (Table 5). Furthermore, the bootstrap validation showed that all the indicators had significant loadings according to the lower and upper confidence intervals (Table 6): For this reason, we considered all of them a good proxy of latent variables.

## 5. Discussion

The hypothesis that sleepiness affects cognition and school performance arises mainly from studies investigating the impact of sleep deprivation/restriction or sleep disorders on cognitive and academic skills. However, despite the fact that sleep disorders and sleep loss induce both sleepiness and cognitive deficits, it is not possible to directly assume that decremented cognitive performances are due to sleepiness. Indeed, sleep loss and sleep disorder may affect brain maturation, cognitive functions, and related brain mechanisms through many different processes (e.g., disruption of sleep related restorative brain processes, oxidative stress, and hypoxia in prefrontal cortex and other brain regions) [2,42,94,95]. Thus, it is possible to stress that, in order to support the idea that there is a relationship between sleepiness and cognition, there is a need of studies that assess directly and specifically the association between sleepiness and cognitive skills [44]. In the present paper, we examined this issue considering many standardized measures of cognitive and academic abilities, also including non-verbal domains, and used also a statistical technique allowing us to explore causal relationships.

The results showed that sleepiness seems to affect several, but not all, cognitive and literacy skills. First of all, we found that sleepiness is related to attention processes. Daytime sleepiness has a detrimental effect on attention and limits the ability to focus and attend to salient information. This result may be considered in accordance with data from other studies showing that sleepiness is associated with processing speed [65] and selective attention in the GO/NO-GO task [63].

Moreover, it seems that sleepiness (perhaps because of reduced attention capacity) may slow learning and may decrease the efficiency of some cognitive and academic skills. With regards to cognitive skills, we found that sleepiness is related only to verbal abilities. Specifically, sleepiness had a negative impact on syntactic comprehension but did not influence *visuo-spatial working memory* and *visuo-constructive skills*. Several studies showed that sleepiness is related to verbal ability and verbal comprehension [57], however, the focus on non-verbal visuo-spatial and visuo-constructive abilities is relatively new [65]. Present data show an unequal vulnerability of verbal vs. non-verbal abilities by sleepiness.

We also found that sleepiness had significant and detrimental effects on some, but not all, literacy skills. In particular, it negatively influenced the orthographic competency, the mathematical abilities, and tended to significantly affect also reading comprehension. At the same time, results showed that sleepiness did not interfere with the efficiency of reading, decoding, and handwriting fluency.

Overall results deserve several comments. Firstly, data clearly show that sleepiness is not related to all cognitive and academic performances. As stated by Beebe and Gonzal [95], though it is tempting to attribute all the daytime difficulties to excessive sleepiness, doing so requires expansion of the already multidimensional construct of sleepiness to include conceptually distinct cognitive function.

Interestingly, we found more profound effects on verbal than non-verbal abilities and on functions requiring executive control with respect to automatized ones. Specifically, sleepiness had a negative impact on oral and written comprehension and orthographic competency but did not influence visuo-spatial working memory, visuo-constructive skills, and handwriting speed. Although the dissociation between verbal and visuo-spatial domains merits some caution, since we did not extensively test all verbal competencies of our children (e.g., verbal working memory and measures of vocabulary, verbal memory are absent), it clearly emerges that sleepiness is unrelated to visual-spatial, constructional, and motor domains of cognition.

With respect to the impact of differences of tasks in terms of automaticity and resource demanding, several studies indicating that sleep loss affects only “higher” cognitive functions related to prefrontal cortex (e.g., creativity, verbal fluency, divergent thinking ability, executive functions, problem solving, and learning new abstract concepts), but it does not influence other automatic and less-complex cognitive processes [4,34,48,96,97,98,99]. For instance, a recent meta-analysis stressed that sleep duration is linked mainly with deficits in higher-order and complex executive functions [4]. Indeed, we found a relation between sleepiness and verbal comprehension (i.e., written and oral/syntactic comprehension), spelling, and such cognitive skills involving complex frontal lobe related verbal, controlled, and meta-cognitive skills, while it did not hamper efficient word decoding and the fast motor activity implied in handwriting.

With respect to reading, after sleep loss and increased sleepiness, the ability such as word decoding may be carried out efficiently through automatic decoding processes [100]. Italian children acquire the decoding process very early and read with a very small rate of errors yet after one year of schooling [101,102,103,104]. In fact, with increasing experience and practice, children progressively pass from the application of an effortful and serial algorithm to a less demanding process based on the fast and automatic retrieval of a memory trace [105,106,107]. With practice, children also automatize the process of grapheme-to-phoneme mapping in a given orthography. This finding could explain why sleepiness is not related to the decoding skills in our sample. Sleep loss may leave automatic cognitive processes (such as reading decoding) unchanged but, at the same time, it continues to affect complex cognitive functions, as highlighted by the partial influence of sleepiness on the reading comprehension skill, that is not an automatic process involving prefrontal controlled skills and meta-cognitive processes and monitoring [108,109,110,111]. Notably, it is important to consider that brain imaging and cognitive studies showed that reading decoding and reading comprehension are dissociable functions [108,111,112,113]. Consistent with our results, Kuroishi et al. [43] found that children with mouth breathing syndrome had poorer performance than controls on the reading comprehension task. However, Ellis et al. [114] found contrasting results: Poor sleepers performed better on the reading comprehension test than medium quality sleepers. To explain their result, Ellis et al. [114] suggested that a compensatory effort can temporarily permit coping with the effect of sleep loss and sleepiness. More generally, Ellis et al. suggested that sleep loss and sleepiness may induce an increase of compensative brain and psychological mechanisms that cope with deficits and, in turn, improve some cognitive skills, thus the performance of sleepy children may be normal or higher [114]. If this was the case, we should have found no relationship between sleepiness and any other skills, while we found that sleepiness affected several cognitive abilities. Of course, we realize that there is a need of more studies that assess the relationship between sleepiness and reading comprehension.

With respect to writing, while spelling is a very demanding and sensitive task, handwriting is considered a fast motor activity. Efficient spelling requires a fine online phonetic-phonological analysis of the acoustic string and correct phoneme-to-grapheme sequential mapping and/or lexical access. As described in the method section, the spelling task used maximized the difficulties of both the mentioned processes, since stimuli were varied for different sources of sublexical spelling difficulties. Coherently, in the literature there are reports of a different sensitivity of spelling vs. reading processes in detecting deficits of automation. For example, adults with compensated dyslexia, as well as adult relatives of dyslexic children, may show spelling errors as a result of their residual learning difficulties [115,116]. Therefore, it seems that spelling, but not reading decoding, may reveal minor learning problems that might otherwise go undetected. Handwriting, instead, became very early fully automated. Handwriting is a rhythmic activity that can reach a frequency of about 5 Hz when accounting for the successive ascending and descending strokes produced by the pen. The high frequency limits the possibility of online control based on sensory information and imposes a mode of control, based on the execution of motor programs. Automation refers to the fact that writing is produced with minimal conscious “effort”, i.e., with minimal attentional and frontal involvement [117,118]. Automation is crucial in handwriting as it allows the allocation of cognitive resources to other processes [119,120].

With regards to the relationships between sleepiness and mathematical skills, our results suggest that sleepiness is linked with all the domains of mathematical competence. One would wonder why also the verbal recall of arithmetical facts, an ability that seems to be more automated, was found to be implied. Accordingly with the neuroscientific and neuropsychological model developed by Dehaene and Cohen [75], the ability of recalling arithmetical facts involves the domain of verbal ability (i.e., not specific for number) implemented within classical frontal and perisylvian language areas of the left hemisphere [75]. In our study, all mathematical domains are involved, confirming the major susceptibility of the verbal component of some mathematical tasks (i.e., arithmetical facts) and probably also the higher reliance on prefrontal related executive attention control of the others [121].

Our study has several limitations. Some studies point out that the relationship between sleepiness and cognitive and scholastic skills is influenced by socioeconomic variables [57]. Moreover, sleepiness may be measured via several methods, including objective methods (such as the multiple sleep latency test, psychomotor vigilance performance, and measures of spontaneous oscillations in pupil diameter). Notably, some studies have stressed that subjective and objective measures of sleepiness are not strictly associated and thus may represent distinct entities that should not be assumed to be equivalent [122]. Thus, other studies are needed to corroborate our data. Nevertheless, the present study clearly showed a relationship between subjective experience of sleepiness and objective neuropsychological measures. In conclusion, we extended the finding about the association between sleepiness and both cognitive and scholastic outcomes. It is highlighted that the presence of subjective sleepiness can be considered a risk factor for cognitive and scholastic difficulties in children, at least in abilities not automatized and in the verbal domain. The present results have clinical and educational implications. In fact, clinicians and educators may consider that sleepy children may not respond optimally to tests or scholastic verifications: It would be useful, if possible, to test children at a different time of day, or different days, when the child is not sleepy and consider sleepiness in formulating conclusions about core reasons for a child′s poor performance. Moreover, comorbidity of sleep problems should be evaluated. Many children with concurrent diagnoses should be reevaluated taking into account the role of sleep in aggravating their performance.

Furthermore, it is important to remember that medical and/or behavioural treatment on sleep disorders (that are related to sleepiness) and, in particular, on sleep habits and hygiene may be in some cases effective and recommended in order to reduce the cognitive difficulties due to sleepiness [5,80,93,113]. Moreover, teachers and health professionals should sensitize and inform the population in order to promote an adequate sleep in children to reduce the impact of sleepiness on cognitive and academic achievement.

## Figures and Tables

**Table 1:** Latent variables and indicators of the model.

Latent Variables	Label	No. of Indicators
Sleepiness	Sleep	1
Mathematical Skills	Math	4
Attention	Att	4
Spelling	Spe	2
Oral Comprehension	Or Comp	1
Reading	Read	2
Visuo-Constructive Abilities	Visc	1
Working Memory	WM	1
Handwriting	Handw	1
Reading Comprehension	Read Comp	1

**Table 2:** Reliability analysis of latent variables.

	Cronbach α	Dillon-Goldstein ρ	1st Eigenvalue	2nd Eigenvalue
Sleepiness	1.00	1.00	1.00	0.00
Mathematical Skills	0.69	0.81	2.08	0.79
Attention	0.84	0.89	2.72	0.52
Spelling	0.82	0.92	1.69	0.31
Oral Comprehension	1.00	1.00	1.00	0.00
Read	0.76	0.89	1.61	0.39
Visuo-Constructive Abilities	1.00	1.00	1.00	0.00
Working Memory	1.00	1.00	1.00	0.00
Handwriting	1.00	1.00	1.00	0.00
Reading Comprehension	1.00	1.00	1.00	0.00

**Table 3:** Outer model with loadings and communalities.

Indicators	Latent Variables	Loadings	Communality
*Sleepiness*	Sleepiness	1.00	1.00
*Computation*	Mathematical Skills	0.69	0.47
*Number Spelling*	Mathematical Skills	0.68	0.47
*Number Ordering*	Mathematical Skills	0.72	0.51
*Arithmetical Facts*	Mathematical Skills	0.78	0.61
*Bell (30 s) 1*	Attention	0.85	0.72
*Bell (30 s) 2*	Attention	0.76	0.57
*Bell (30 s) 3*	Attention	0.89	0.80
*Bell (30 s) 4*	Attention	0.77	0.60
*Regular*	Spelling	0.93	0.87
*Pseudowords*	Spelling	0.87	0.76
*Oral Comprehension*	Oral Comprehension	1.00	1.00
*Text Comprehension*	Reading Comprehension	1.00	1.00
*Reading Speed*	Reading	0.92	0.85
*Reading Accuracy*	Reading	0.87	0.76
*Rey*	Visuo-Constructive Abilities	1.00	1.00
*VPTA*	Working Memory	1.00	1.00
*Handwriting Speed*	Handwriting	1.00	1.00

**Table 4:** Cross-loadings of the indicators. Indicators of each latent variable were reported in bold.

Indicators	SLE	MAT	ATT	SPE	OR COMP	READ	VISC	WM	HANDW	READ COMP
Sleepiness	**1.00**	−0.18	−0.22	−0.18	−0.25	−0.11	−0.02	−0.06	−0.12	−0.14
Computation	−0.10	**0.69**	0.30	0.41	0.38	0.35	0.36	0.22	0.26	0.16
Number Spelling	−0.14	**0.68**	0.06	0.21	0.31	0.27	0.21	0.29	0.13	0.06
Number Ordering	−0.11	**0.72**	0.09	0.35	0.33	0.31	0.44	0.24	0.21	0.07
Arithmetical Facts	−0.14	**0.78**	0.18	0.28	0.35	0.40	0.40	0.45	0.26	0.21
Bell (30 s) 1	−0.22	0.18	**0.85**	0.06	0.24	0.20	0.04	0.07	0.28	−0.03
Bell (30 s) 2	−0.10	0.14	**0.76**	0.10	0.13	0.14	0.16	0.04	0.26	−0.03
Bell (30 s) 3	−0.21	0.19	**0.90**	0.10	0.17	0.26	0.10	0.03	0.26	0.09
Bell (30 s) 4	−0.14	0.16	**0.77**	0.12	0.09	0.12	0.15	0.04	0.16	−0.10
Regular	−0.18	0.35	0.09	**0.93**	0.16	0.33	0.30	0.12	0.16	0.12
Pseudowords	−0.15	0.43	0.11	**0.90**	0.20	0.38	0.23	0.20	0.16	0.07
Oral Comprehension	−0.25	0.47	0.20	0.20	**1.00**	0.30	0.24	0.26	0.19	0.29
Reading Speed	−0.11	0.36	0.24	0.28	0.25	**0.92**	0.06	0.13	0.34	0.29
Reading Accuracy	−0.09	0.50	0.16	0.43	0.29	**0.87**	0.07	0.31	0.29	0.31
Rey	−0.02	0.48	0.12	0.29	0.24	0.07	**1.00**	0.29	0.26	0.01
VPTA	−0.06	0.43	0.05	0.17	0.26	0.23	0.29	**1.00**	0.17	0.07
Handwriting Speed	−0.12	0.29	0.29	0.17	0.19	0.35	0.26	0.17	**1.00**	0.23
Text Comprehension	−0.14	0.18	−0.01	0.11	0.29	0.34	0.01	0.07	0.23	**1.00**

**Table 5:** Bootstrap validation of inner model paths coefficient.

Relation	Original	Mean Bootstrap	Std. Error	Lower CI	Upper CI
SLE ≥ MATH	−0.1759	−0.2023	0.0801	−0.337	−0.0626
SLE ≥ ATT	−0.2177	−0.2363	0.0768	−0.373	−0.1079
SLE ≥ SPE	−0.1828	−0.1861	0.0665	−0.304	−0.0603
SLE ≥ OR COMP	−0.2510	−0.2501	0.0703	−0.376	−0.1122
SLE ≥ Read	−0.1132	−0.1233	0.0929	−0.275	0.1083
SLE ≥ VISC	−0.0240	−0.0219	0.0803	−0.178	0.1388
SLE ≥ WM	−0.0631	−0.0614	0.0786	−0.212	0.0928
SLE ≥ HANDW	−0.1173	−0.1176	0.0784	−0.267	0.0338
SLE ≥ READ COMP	−0.1376	−0.1382	0.0927	−0.315	0.0352

**Table 6:** Bootstrap validation of loadings between indicators and latent variables (outer model).

	Original	Mean Bootstrap	Std. Error	Lower CI	Upper CI
SLE-Sleepiness	1.000	1.000	0.000	1.000	1.000
MATH-Computation	0.686	0.630	0.021	0.061	0.897
MATH-Number spelling	0.682	0.647	0.019	0.199	0.912
MATH-Number ordering	0.716	0.653	0.018	0.132	0.871
MATH-Arithmetical facts	0.782	0.721	0.017	0.304	0.914
ATT-Bell (30 s) 1	0.846	0.835	0.009	0.688	0.929
ATT-Bell (30 s) 1	0.756	0.720	0.013	0.350	0.851
ATT-Bell (30 s) 1	0.895	0.876	0.008	0.727	0.939
ATT-Bell (30 s) 1	0.774	0.753	0.010	0.521	0.871
SPE-Regular	0.934	0.914	0.014	0.745	0.994
SPE-Pseudowords	0.904	0.880	0.013	0.631	0.955
OR COMP -Oral comprehension	1.000	1.000	0.000	1.000	1.000
READ-Reading speed	0.921	0.835	0.027	0.048	0.999
READ-Reading accuracy	0.872	0.796	0.027	0.094	0.994
VISC-Rey	1.000	1.000	0.000	1.000	1.000
WM-VPTA	1.000	1.000	0.000	1.000	1.000
HANDW-Handwriting Speed	1.000	1.000	0.000	1.000	1.000
READ COMP-Text Comprehension	1.000	1.000	0.000	1.000	1.000
