# Erratum: Macchitella et al. Sleepiness, Neuropsychological Skills, and Scholastic Learning in Children. *Brain Sci*. 2020, *10*, 529

**DOI:** 10.3390/brainsci11070892

**Published:** 2021-07-06

**Authors:** Luigi Macchitella, Chiara Valeria Marinelli, Fulvio Signore, Enrico Ciavolino, Paola Angelelli

**Affiliations:** Department of History, Society and Human Studies, Lab of Applied Psychology and Intervention, University of Salento, 73100 Lecce, Italy; luigi.macchitella@unisalento.it (L.M.); fulvio.signore@unisalento.it (F.S.); enrico.ciavolino@unisalento.it (E.C.); paola.angelelli@unisalento.it (P.A.)

The author wishes to make an erratum to the published version of the paper [1]. The citation order was mistaken in the production process and three references needed to be added to the References section; thus, the reference list needs to be updated. Moreover, in Section 2.3.3. of the main text, the correct reference for the AC-MT battery is [73]; in the discussion, the correct reference for Beebe and Gonzal is [94]; and in the final paragraph, “Furthermore, it is important to remember that medical and/or behavioural treatment on sleep disorders (that are related to sleepiness) and, in particular, on sleep habits and hygiene may be in some cases effective and recommended in order to reduce the cognitive difficulties due to sleepiness”, the correct references are [5,123–125].

The correct reference list is listed as below:

1. Curcio, G.; Ferrara, M.; De Gennaro, L. Sleep loss, learning capacity and academic performance. *Sleep Med. Rev.* **2006**, *10*, 323–337.
2. Sadeh, A. Consequences of Sleep Loss or Sleep Disruption in Children. *Sleep Med. Clin.* **2007**, *2*, 513–520.
3. Beebe, D.W. Cognitive, behavioral, and functional consequences of inadequate sleep in children and adolescents. *Pediatr. Clin.* **2011**, *58*, 649–665.
4. Astill, R.G.; Van der Heijden, K.B.; Van IJzendoorn, M.H.; Van Someren, E.J. Sleep, cognition, and behavioral problems in school-age children: A century of research meta-analyzed. *Psychol. Bull.* **2012**, *138*, 1109.
5. Hershner, S.D.; Chervin, R.D. Causes and consequences of sleepiness among college students. *Nat. Sci. Sleep* **2014**, *6*, 73–84.
6. De Bruin, E.J.; Van Run, C.; Staaks, J.; Meijer, A.M. Effects of sleep manipulation on cognitive functioning of adolescents: A systematic review. *Sleep Med. Rev.* **2017**, *32*, 45–57.
7. Tham, E.; Schneider, N.; Broekman, B.F.P. Infant sleep and its relation with cognition and growth: A narrative review. *Nat. Sci. Sleep* **2017**, *9*, 135–149.
8. Reynaud, E.; Vecchierini, M.-F.; Heude, B.; Charles, M.A.; Plancoulaine, S. Sleep and its relation to cognition and behaviour in preschool-aged children of the general population: A systematic review. *J. Sleep Res.* **2018**, 27, e12636.
9. Morse, A.M.; Kothare, S.V. Evaluation and Management of a Sleepy Child. In *Allergy and Sleep*; Springer: Berlin/Heidelberg, Germany, 2019; pp. 87–104.
10. Blunden, S.; Hoban, T.F.; Chervin, R.D. Sleepiness in children. *Sleep Med. Clin.* **2006**, *1*, 105–118.
11. Owens, J.A.; Babcock, D.; Weiss, M. Evaluation and Treatment of Children and Adolescents with Excessive Daytime Sleepiness. *Clin. Pediatr.* **2020**, *59*, 340–351.
12. Moore, M.; Meltzer, L.J. The sleepy adolescent: Causes and consequences of sleepiness in teens. *Paediatr. Respir. Rev.* **2008**, *9*, 114–121.
13. Fallone, G.; Owens, J.A.; Deane, J. Sleepiness in children and adolescents: Clinical implications. *Sleep Med. Rev*. **2002**, *6*, 287–306.
14. Crowley, S.J.; Wolfson, A.R.; Tarokh, L.; Carskadon, M.A. An update on adolescent sleep: New evidence informing the perfect storm model. *J. Adolesc.* **2018**, *67*, 55–65.
15. Carskadon, M.A.; Wolfson, A.R.; Acebo, C.; Tzischinsky, O.; Seifer, R. Adolescent sleep patterns, circadian timing, and sleepiness at a transition to early school days. *Sleep* **1998**, *21*, 871–881.
16. Jenni, O.G.; LeBourgeois, M.K. Understanding sleep-wake behavior and sleep disorders in children: The value of a model. *Curr. Opin. Psychiatry* **2006**, *19*, 282–287.
17. Carskadon, M.A. Sleep in Adolescents: The Perfect Storm. *Pediatr. Clin. N. Am*. **2011**, *58*, 637–647.
18. Minges, K.E.; Redeker, N.S. Delayed school start times and adolescent sleep: A systematic review of the experimental evidence. *Sleep Med. Rev.* **2016**, *28*, 86–95.
19. Marx, R.; Tanner-Smith, E.; Davison, C.M.; Ufholz, L.-A.; Freeman, J.; Shankar, R.; Newton, L.; Brown, R.S.; Parpia, A.S.; Cozma, I.; et al. Later school start times for supporting the education, health, and well-being of high school students. *Cochrane Database Syst. Rev.* **2017**, *7*, 1–57.
20. Gibson, E.S.; Powles, A.C.P.; Thabane, L.; O’Brien, S.; Molnar, D.S.; Trajanovic, N.; Ogilvie, R.; Shapiro, C.M.; Yan, M.; Chilcott-Tanser, L. “Sleepiness” is serious in adolescence: Two surveys of 3235 Canadian students. *BMC Public Health* **2006**, *6*, 116.
21. Roehrs, T.; Roth, T. Caffeine: Sleep and daytime sleepiness. *Sleep Med. Rev.* **2008**, *12*, 153–162.
22. Calamaro, C.J.; Mason, T.B.; Ratcliffe, S.J. Adolescents living the 24/7 lifestyle: Effects of caffeine and technology on sleep duration and daytime functioning. *Pediatrics* **2009**, *123*, 1005.
23. Higuchi, S.; Motohashi, Y.; Liu, Y.; Maeda, A. Effects of playing a computer game using a bright display on presleep physiological variables, sleep latency, slow wave sleep and REM sleep. *J. Sleep Res.* **2005**, *14*, 267–273.
24. Joo, S.; Shin, C.; Kim, J.; Yi, H.; Ahn, Y.; Park, M.; Kim, J.; Lee, S. Prevalence and correlates of excessive daytime sleepiness in high school students in Korea. *Psychiatry Clin. Neurosci*. **2005**, *59*, 433–440.
25. Foley, L.S.; Maddison, R.; Jiang, Y.; Marsh, S.; Olds, T.S.; Ridley, K. Presleep Activities and Time of Sleep Onset in Children. *Pediatrics* **2013**, *131*, 276–282.
26. Liu, Y.; Zhang, J.; Li, S.X.; Chan, N.Y.; Yu, M.W.M.; Lam, S.P.; Chan, J.; Li, A.M.; Wing, Y.-K. Excessive daytime sleepiness among children and adolescents: Prevalence, correlates, and pubertal effects. *Sleep Med.* **2019**, *53*, 1–8.
27. Beyens, I.; Nathanson, A.I. Electronic media use and sleep among preschoolers: Evidence for time-shifted and less consolidated sleep. *Health Commun.* **2019**, *34*, 537–544.
28. Crowley, S.J.; Acebo, C.; Carskadon, M.A. Sleep, circadian rhythms, and delayed phase in adolescence. *Sleep Med.* **2007**, *8*, 602–612.
29. Altena, E.; Baglioni, C.; Espie, C.A.; Ellis, J.; Gavriloff, D.; Holzinger, B.; Schlarb, A.; Frase, L.; Jernelöv, S.; Riemann, D. Dealing with sleep problems during home confinement due to the COVID-19 outbreak: Practical recommendations from a task force of the European CBT-I Academy. *J. Sleep Res.* **2020**, *29*, e13052.
30. Buheji, M.; Hassani, A.; Ebrahim, A.; da Costa Cunha, K.; Jahrami, H.; Baloshi, M.; Hubail, S. Children and Coping During COVID-19: A Scoping Review of Bio-Psycho-Social Factors. *Int. J. Appl.* **2020**, *10*, 8–15.
31. Xiang, M.; Zhang, Z.; Kuwahara, K. Impact of COVID-19 pandemic on children and adolescents’ lifestyle behavior larger than expected. *Prog. Cardiovasc. Dis.* **2020**, *63*, 531.
32. King, D.L.; Delfabbro, P.H.; Billieux, J.; Potenza, M.N. Problematic online gaming and the COVID-19 pandemic. *J.Behav. Addict.* **2020**, *9*, 184–186.
33. Lepido, D.; Rolander, N. Housebound Italian Kids Strain Network with Fortnite Marathon. 2020. Available online: https://www.bloomberg.com/news/articles/2020-03-12/housebound-italian-kids-strain-networkwith-fortnite-marathon (accessed on 12 March 2020).
34. Sadeh, A.; Gruber, R.; Raviv, A. Sleep, neurobehavioral functioning, and behavior problems in school-age children. *Child Dev.* **2002**, *73*, 405–417.
35. Steenari, M.R.; Vuontela, V.; Paavonen, E.J.; Carlson, S.; Fjallberg, M.; Aronen, E.T. Working memory and sleep in 6- to 13-year-old schoolchildren. *J. Am. Acad. Child Adolesc. Psychiatry* **2003**, *42*, 85–92.
36. Gottlieb, D.J.; Chase, C.; Vezina, R.M.; Heeren, T.; Corwin, M.J.; Auerbach, S.; Weese-Mayer, D.E.; Lesko, S.M. Sleep-disordered breathing symptoms are associated with poorer cognitive function in 5-year-old children. *J. Pediatr.* **2004**, *145*, 458–464.
37. O’Brien, L.M.; Mervis, C.B.; Holbrook, C.R.; Bruner, J.L.; Smith, N.H.; McNally, N.; McClimment, M.C.; Gozal, D. Neurobehavioral correlates of sleep-disordered breathing in children. *J. Sleep Res.* **2004**, *13*, 165–172.
38. Gradisar, M.; Terrill, G.; Johnston, A.; Douglas, P. Adolescent sleep and working memory performance. *Sleep Biol. Rhythm*. **2008**, *6*, 146–154.
39. Goodlin-Jones, B.; Tang, K.; Liu, J.; Anders, T.F. Sleep problems, sleepiness and daytime behavior in preschool-age children. *J. Child Psychol. Psychiatry* **2009**, *50*, 1532–1540.
40. Kopasz, M.; Loessl, B.; Hornyak, M.; Riemann, D.; Nissen, C.; Piosczyk, H.; Voderholzer, U. Sleep and memory in healthy children and adolescents-a critical review. *Sleep Med. Rev.* **2010**, *14*, 167–177.
41. Bourke, R.; Anderson, V.; Yang, J.S.; Jackman, A.R.; Killedar, A.; Nixon, G.M.; Davey, M.J.; Walker, A.M.; Trinder, J.; Horne, R.S.C. Cognitive and academic functions are impaired in children with all severities of sleep-disordered breathing. *Sleep Med.* **2011**, *12*, 489–496.
42. Esposito, M.; Antinolfi, L.; Gallai, B.; Parisi, L.; Roccella, M.; Marotta, R.; Lavano, S.M.; Mazzotta, G.; Precenzano, F.; Carotenuto, M. Executive dysfunction in children a_ected by obstructive sleep apnea syndrome: An observational study. *Neuropsychiatr. Dis. Treat.* **2013**, *9*, 1087–1094.
43. Kuroishi, R.C.S.; Garcia, R.B.; Valera, F.C.P.; Anselmo-Lima, W.T.; Fukuda, M.T.H. Deficits in working memory, reading comprehension and arithmetic skills in children with mouth breathing syndrome: Analytical cross-sectional study. *São Paulo Med. J.* **2015**, *133*, 78–83.
44. Liu, J.; Liu, X.; Ji, X.; Wang, Y.; Zhou, G.; Chen, X. Sleep disordered breathing symptoms and daytime sleepiness are associated with emotional problems and poor school performance in children. *Psychiatry Res.* **2016**, *242*, 218–225.
45. Hunter, S.J.; Gozal, D.; Smith, D.L.; Philby, M.F.; Kaylegian, J.; Kheirandish-Gozal, L. Effect of Sleep-disordered Breathing Severity on Cognitive Performance Measures in a Large Community Cohort of Young School-aged Children. *Am. J. Respir. Crit. Care Med.* **2016**, *194*, 739–747.
46. Ruberto, M.; Precenzano, F.; Parisi, L.; Salerno, M.; Maltese, A.; Messina, G.; Roccella, M. Visuomotor integration skills in children a_ected by obstructive sleep apnea syndrome: A case-control study. *Acta Med. Mediterr.* **2016**, *32*, 1659–1663.
47. Randazzo, A.C.; Schweitzer, P.K.; Walsh, J.K. Cognitive function following 3 nights of sleep restriction in home monitoring. *Sleep* **1998**, *21*, s249.
48. Randazzo, A.C.; Muehlbach, M.J.; Schweitzer, P.K.; Waish, J.K. Cognitive function following acute sleep restriction in children ages 10–14. *Sleep* **1998**, *21*, 861–868.
49. Sadeh, A.; Gruber, R.; Raviv, A. The effects of sleep restriction and extension on school-age children: What a difference an hour makes. *Child Dev.* **2003**, *74*, 444–455.
50. Beebe, D.W.; Fallone, G.; Godiwala, N.; Flanigan, M.; Martin, D.; Schaffner, L.; Amin, R. Feasibility and behavioral effects of an at-home multi-night sleep restriction protocol for adolescents. *J. Child. Psychol. Psychiatry* **2008**, *49*, 915–923.
51. Jiang, F.; VanDyke, R.D.; Zhang, J.; Li, F.; Gozal, D.; Shen, X. Effect of chronic sleep restriction on sleepiness and working memory in adolescents and young adults. *J. Clin. Exp. Neuropsychol.* **2011**, *33*, 892–900.
52. Lo, J.C.; Ong, J.L.; Leong, R.L.; Gooley, J.J.; Chee, M.W. Cognitive Performance, Sleepiness, and Mood in Partially Sleep Deprived Adolescents: The Need for Sleep Study. *Sleep* **2016**, *39*, 687–698.
53. Fallone, G.; Acebo, C.; Arnedt, J.T.; Seifer, R.; Carskadon, M.A. Effects of acute sleep restriction on behavior, sustained attention, and response inhibition in children. *Percept. Mot. Skills* **2001**, *93*, 213–229.
54. Fallone, G.; Acebo, C.; Seifer, R.; Carskadon, M.A. Experimental restriction of sleep opportunity in children: Effect on teacher rating. *Sleep* **2005**, *28*, 1561–1567.
55. Chuah, L.Y.M.; Chee, M.W.L. Cholinergic Augmentation Modulates Visual Task Performance in Sleep-Deprived Young Adults. *J. Neurosci.* **2008**, *28*, 11369–11377.
56. Drake, C.L.; Nickel, C.; Burduvali, E.; Roth, T.; Jefferson, C.; Badia, P. The Pediatric Daytime Sleepiness Scale (PDSS): Sleep Habits and School Outcomes in Middle-school Children. *Sleep* **2003**, *26*, 455–458.
57. Buckhalt, J.A.; Keller, P.; El-Sheikh, M. Children’s Sleep and Cognitive Functioning: Race and Socioeconomic Status as Moderators of Effects. *Child Dev*. **2007**, *78*, 213–231.
58. Perez-Chada, D.; Perez-Lloret, S.; Videla, A.J.; Cardinali, D.; Bergna, M.A.; Fernández-Acquier, M.; Larrateguy, L.; Zabert, G.E.; Drake, C.L. Sleep Disordered Breathing and Daytime Sleepiness Are Associated with Poor Academic Performance In Teenagers. A Study Using the Pediatric Daytime Sleepiness Scale (PDSS). *Sleep* **2007**, *30*, 1698–1703.
59. Anderson, B.; Storfer-Isser, A.; Taylor, H.G.; Rosen, C.L.; Redline, S. Associations of executive function with sleepiness and sleep duration in adolescents. *Pediatrics* **2009**, *123*, e701–e707.
60. Buckhalt, J.A.; Keller, P.S.; Kelly, R.J.; El-Sheikh, M. Concurrent and Longitudinal Relations between Children’s Sleep and Cognitive Functioning: The Moderating Role of Parent Education. *Child Dev.* **2009**, *80*, 875–892.
61. Dewald, J.F.; Meijer, A.M.; Oort, F.J.; Kerkhof, G.A.; Bögels, S.M. The influence of sleep quality, sleep duration and sleepiness on school performance in children and adolescents: A meta-analytic review. *Sleep Med. Rev.* **2010**, *14*, 179–189.
62. Buckhalt, J.A. Children’s Sleep, Sleepiness, and Performance on Cognitive Tasks. *WMF Press Bull*. **2011**, *2*, 1–12.
63. Cerasuolo, M.; Giganti, F.; Conte, F.; Costanzo, L.M.; Della Monica, C.; Arzilli, C.; Marchesano, R.; Perrella, A.; Ficca, G. Schooltime subjective sleepiness and performance in Italian primary school children. *Chronol. Int.* **2016**, *33*, 1–10.
64. Bub, K.L.; Buckhalt, J.A.; El-Sheikh, M. Children’s sleep and cognitive performance: A cross-domain analysis of change over time. *Dev. Psychol.* **2011**, *47*, 1504–1514.
65. Calhoun, S.L.; Fernandez-Mendoza, J.; Vgontzas, A.N.; Mayes, S.D.; Tsaoussoglou, M.; Rodriguez-Muñoz, A.; Bixler, E.O. Learning, Attention/Hyperactivity, and Conduct Problems as Sequelae of Excessive Daytime Sleepiness in a General Population Study of Young Children. *Sleep* **2012**, *35*, 627–632.
66. Giannotti, F.; Cortesi, F.; Ottaviano, S. Sleep pattern daytime functioning and school performance in adolescence: Preliminary data on an Italian representative sample. *Sleep Res.* **1997**, *26*, 196.
67. Bruni, O.; Ferini-Strambi, L.; Russo, P.M.; Antignani, M.; Innocenzi, M.; Ottaviano, P.; Valente, D.; Ottaviano, S. Sleep disturbances and teacher ratings of school achievement and temperament in children. *Sleep Med.* **2006**, *7*, 43–48.
68. Shin, C.; Kim, J.; Lee, S.; Ahn, Y.; Joo, S. Sleep habits, excessive daytime sleepiness and school performance in high school students. *Psychiatry Clin. Neurosci*. **2003**, *57*, 451–453.
69. Pruneti, C. Aggiornamento della standardizzazione italiana del test delle Matrici Progressive Colorate di Raven. *Boll. Psicol. Appl*. **1996**, *217*, 51–57.
70. Lewandowski, A.S.; Toliver-Sokol, M.; Palermo, T.M. Evidence-Based Review of Subjective Pediatric Sleep Measures. *J. Pediatr. Psychol.* **2011**, *36*, 780–793.
71. Cornoldi, C.; Carretti, B. *Prove MT-3- Clinica. Florence*; Organizzazioni Speciali: Florence, Italy, 2016.
72. Angelelli, P.; Marinelli, C.V.; Iaia, M.; Notarnicola, A.; Costabile, D.; Judica, A.; Zoccolot-ti, P.; Luzzatti, C. *DDO 2: Diagnosi dei Disturbi Ortografici in età Evolutiva*; Edizioni Centro Studi Erickson: Trento, Italy, 2016.
73. Cornoldi, C.; Lucangeli, D.; Bellina, M. *AC-MT 6-11. Test per la Valutazione delle Abilità di Calcolo e Risoluzione dei Problem;* Edizioni Centro Studi Erickson: Trento, Italy, 2012.
74. McCloskey, M.; Caramazza, A.; Basili, A. Cognitive mechanisms in number processing and calculation: Evidence from dyscalculia. *Brain Cogn.* **1985**, *4*, 171–196.
75. Dehaene, S.; Cohen, L. Cerebral pathways for calculation: Double dissociation between rote verbal and quantitative knowledge of arithmetic. *Cortex* **1997**, *33*, 219–250.
76. Bisiacchi, P.S.; Cendron, M.; Gugliotta, M.; Tressoldi, P.; Vio, C. *BVN5-11. Batteria di Valutazione Neuropsicologica per l’età Evolutiva*; Edizioni Erickson: Trento, Italy, 2005.
77. Hamstra-Bletz, L.; Blöte, A.W. A Longitudinal Study on Dysgraphic Handwriting in Primary School. *J. Learn. Disabil.* **1993**, *26*, 689–699.
78. Mammarella, I.C.; Toso, C.; Pazzaglia, F.; Cornoldi, C. BVS-Corsi: *The Corsi Blocks Task and the BVS Battery for Visuospatial Memory Assessment*; Edizioni Centro Studi Erickson: Trento, Italy, 2008.
79. Cornoldi, C.; Rigoni, F.; Venneri, A.; Vecchi, T. Passive and active processes in visuo-spatial memory: Double dissociation in developmental learning disabilities. *Brain Cogn.* **2000**, *43*, 17–20.
80. Mammarella, I.C.; Cornoldi, C.; Pazzaglia, F.; Toso, C.; Grimoldi, M.; Vio, C. Evidence for a double dissociation between spatial-simultaneous and spatial-sequential working memory in visuospatial (nonverbal) learning disabled children. *Brain Cogn.* **2006**, *62*, 58–67.
81. Gauthier, L.; Dehaut, F.; Joanette, Y. The Bells test: A quantitative and qualitative test for visual neglect. *Int. J. Clin. Psychol.* **1989**, *11*, 49–54.
82. Rey, A. L’examen psychologique dans les cas d’encephalopathie traumatique. *Arch. Psychol.* **1941**, *28*, 286–340.
83. Akshoomo, N.A.; Stiles, J. Developmental trends in visuospatial analysis and planning: I. copying a complex figure. *Neuropsychology* **1995**, *9*, 364–377.
84. Shin, M.S.; Park, S.-Y.; Park, S.-R.; Seol, S.-H.; Kwon, J.S. Clinical and empirical applications of the Rey-Osterrieth Complex Figure Test. *Nat. Protoc.* **2006**, *1*, 892–899.
85. Strauss, E.; Sherman, E.M.; Spreen, O. *A Compendium of Neuropsychological Tests: Administration, Norms, and Commentary*; American Chemical Society: Washington, DC, USA, 2006.
86. Ferrara-Mori, G. Traduzione Italiana del Manuale del Centre de Psychologie Appliquèe; Organizzazioni Speciali: Florence, Italy. Available online: https://opac.sbn.it/opacsbn/opaclib?db=solr_iccu&resultForward=opac/iccu/brief.jsp&from=1&nentries=10&searchForm=opac/iccu/error.jsp&do_cmd=search_show_cmd&item:5032:Nomi::@frase@=IT%5CICCU%5CSBLV%5C000277 (accessed on 7 August 2020).
87. Sanchez, G. *PLS Path Modeling with R*; Trowchez Editions: Berkeley, CA, USA, 2013.
88. Ciavolino, E. General distress as second order latent variable estimated through PLS-PM approach. *Electron. J. Appl. Stat. Anal.* **2012**, *5*, 458–464.
89. Ciavolino, E.; Carpita, M.; Nitti, M. High-order PLS path model with qualitative external information. *Qual. Quant.* **2015**, *49*, 1609–1620.
90. Signore, F.; Catalano, A.; De Carlo, E.; Madaro, A.; Ingusci, E. The role of employability in students during academic experience: A preliminary study through PLS-PM technique. *Electron. J. Appl. Stat. Anal*. **2019**, *12*, 720–747.
91. Cheah, J.H.; Ting, H.; Ramayah, T.; Memon, M.A.; Cham, T.H.; Ciavolino, E. A comparison of five reflective–formative estimation approaches: Reconsideration and recommendations for tourism research. *Qual. Quant.* **2018**, *53*, 1421–1458.
92. Hair, J.F., Jr.; Hult, G.T.M.; Ringle, C.; Sarstedt, M. *A Primer on Partial Least Squares Structural Equation Modeling (PLS-SEM)*; Sage Publications: Sauzend Oaks, CA, USA, 2016.
93. Svingos, A.; Greif, S.; Bailey, B.; Heaton, S. The Relationship between Sleep and Cognition in Children Referred for Neuropsychological Evaluation: A Latent Modeling Approach. *Children* **2018**, *5*, 33.
94. Beebe, D.W.; Gozal, D. Obstructive sleep apnea and the prefrontal cortex: Towards a comprehensive model linking nocturnal upper airway obstruction to daytime cognitive and behavioral deficits. *J. Sleep Res.* **2002**, *11*, 1–16.
95. Alkadhi, K.; Zagaar, M.; Alhaider, I.; Salim, S.; Aleisa, A. Neurobiological consequences of sleep deprivation. *Curr. Neuropharmacol*. **2013**, *11*, 231–249.
96. Horne, J.A. Sleep loss and divergent thinking ability. *Sleep* **1988**, *11*, 528–536.
97. Harrison, Y.; Horne, J. Sleep loss impairs short and novel language tasks having a prefrontal focus. *J. Sleep Res.* **1998**, *7*, 95–100.
98. Harrison, Y.; Horne, J.; Rothwell, A. Prefrontal neuropsychological effects of sleep deprivation in young adults-a model for healthy aging? *Sleep* **2000**, *23*, 1–7.
99. Jennings, J.R.; Monk, T.H.; Van der Molen, M.W. Sleep deprivation influences some but not all processes of supervisory attention. *Psychol. Sci.* **2003**, *14*, 473–486.
100. Swann, C.E.; Yelland, G.W.; Redman, J.R.; Rajaratnam, S.M. Chronic partial sleep loss increases the facilitatory role of a masked prime in a word recognition task. *J. Sleep Res*. **2006**, *15*, 23–29.
101. Tressoldi, P.; Stella, G.; Faggella, M. The Development of Reading Speed in Italians with Dyslexia. *J. Learn. Disabil*. **2001**, *34*, 414–417.
102. Orsolini, M.; Fanari, R.; Serra, G.; Cioce, R.; Rotondi, A.; Dassisti, A.; Maronato, C. Primi progressi nell’apprendimento della lettura: Una riconsiderazione del ruolo della consapevolezza fonologica. *Psicol. Clin. Sviluppo*. **2003**, *7*, 403–436.
103. Zoccolotti, P.; De Luca, M.; Di Pace, E.; Gasperini, F.; Judica, A.; Spinelli, D. Word length effect in early reading and in developmental dyslexia. *Brain Lang.* **2005**, *93*, 369–373.
104. Marinelli, C.V.; Romani, C.; Burani, C.; McGowan, V.A.; Zoccolotti, P. Costs and Benefits of Orthographic Inconsistency in Reading: Evidence from a Cross-Linguistic Comparison. *PLoS ONE* **2016**, *11*, e0157457.
105. Logan, G.D. Toward an instance theory of automatization. *Psychol. Rev.* **1988**, *95*, 492.
106. Marinelli, C.V.; Cellini, P.; Zoccolotti, P.; Angelelli, P. Lexical processing and distributional knowledge in sound–spelling mapping in a consistent orthography: A longitudinal study of reading and spelling in dyslexic and typically developing children. *Cogn. Neuropsychol.* **2017**, *34*, 163–186.
107. Zoccolotti, P.; De Luca, M.; Marinelli, C.V.; Spinelli, D. Predicting individual differences in reading, spelling, and maths in a sample of typical developing children: A study in the perspective of comorbidity. *PLoS ONE* **2020**, *15*, e0231937.
108. Cain, K.; Oakhill, J. Reading Comprehension Difficulties. In *Handbook of Children’s Literacy*; Springer Science and Business Media LLC: Berlin/Heidelberg, Germany, 2004; pp. 313–338.
109. Van Kraayenoord, C.E. The role of metacognition in reading comprehension. *J. Pendidik. Bhs*. **2010**, *6*, 277–302.
110. Moss, J.; Schunn, C.D.; Schneider, W.; McNamara, D.S.; VanLehn, K. The neural correlates of strategic reading comprehension: Cognitive control and discourse comprehension. *NeuroImage* **2011**, *58*, 675–686.
111. Patael, S.Z.; Farris, E.A.; Black, J.M.; Hancock, R.; Gabrieli, J.D.E.; Cutting, L.E.; Hoeft, F. Brain basis of cognitive resilience: Prefrontal cortex predicts better reading comprehension in relation to decoding. *PLoS ONE* **2018**, *13*, e0198791.
112. Zoccolotti, P.; De Luca, M.; Di Pace, E.; Judica, A.; Orlandi, M.; Spinelli, D. Markers of developmental surface dyslexia in a language (Italian) with high grapheme–phoneme correspondence. *Appl. Psycholinguist.* **1999**, *20*, 191–216.
113. Judica, A.; De Luca, M.; Spinelli, D.; Zoccolotti, P. Training of developmental surface dyslexia improves reading performance and shortens eye fixation duration in reading. *Neuropsychol. Rehabil.* **2002**, *12*, 177–197.
114. Ellis, S.K.; Walczyk, J.J.; Buboltz, W.; Felix, V. The relationship between self-reported sleep quality and reading comprehension skills. *Sleep Sci.* **2014**, *7*, 189–196.
115. Bruck, M. Component spelling skills of college students with childhood diagnoses of dyslexia. *Learn. Disabil. Q.* **1993**, *16*, 171–184.
116. Wolff, P.H.; Melngailis, I.; Kotwica, K. Family patterns of developmental dyslexia part III: Spelling errors as behavioral phenotype. *Am. J. Med. Genet.* **1996**, *67*, 378–386.
117. Hasher, L.; Zacks, R.T. Automatic and effortful processes in memory. *J. Exp. Psychol. Gen.* **1979**, *108*, 356.
118. Tucha, O.; Tucha, L.; Lange, K.W. Graphonomics, automaticity and handwriting assessment. *Literacy* **2008**, *42*, 145–155.
119. Berninger, V.W.; Swanson, H.L. Modifying Hayes and Flower’s model of skilled writing to explain beginning and developing writing. *Adv. Cogn. Educ. Pract.* **1994**, *2*, 57–81.
120. Jones, D.; Christensen, C.A. Relationship between automaticity in handwriting and students’ ability to generate written text. *J. Educ. Psychol.* **1999**, *91*, 44.
121. Arsalidou, M.; Taylor, M.J. Is 2 + 2 = 4? Meta-analyses of brain areas needed for numbers and calculations. *Neuroimage* **2011**, *54*, 2382–2393.
122. Franzen, P.L.; Siegle, G.J.; Buysse, D.J. Relationships between affect, vigilance, and sleepiness following sleep deprivation. *J. Sleep Res*. **2008**, *17*, 34–41.
123. Owens, L.J.; France, K.G.; Wiggs, L. Review Article: Behavioural and cognitive-behavioural interventions for sleep disorders in infants and children: A review. *Sleep Med. Rev*. **1999**, *3*, 281–302.
124. Marcus, C.L.; Radcliffe, J.; Konstantinopoulou, S.; Beck, S.E.; Cornaglia, M.A.; Traylor, J.; DiFeo, N.; Karamessinis, L.R.; Gallagher, P.R.; Meltzer, L.J. Effects of Positive Airway Pressure Therapy on Neurobehavioral Outcomes in Children with Obstructive Sleep Apnea. *Am. J. Respir. Crit. Care Med.* **2012**, *185*, 998–1003.
125. Taylor, H.G.; Bowen, S.R.; Beebe, D.W.; Hodges, E.; Amin, R.; Arens, R.; Chervin, R.D.; Garetz, S.L.; Katz, E.S.; Moore, R.H.; et al. Cognitive Effects of Adenotonsillectomy for Obstructive Sleep Apnea. *Pediatrics* **2016**, *138*, e20154458.

The authors and the Editorial Office apologize for any inconvenience this may cause to all readers.
